# Is RAD-seq suitable for phylogenetic inference? An in silico assessment and optimization

**DOI:** 10.1002/ece3.512

**Published:** 2013-02-27

**Authors:** Marie Cariou, Laurent Duret, Sylvain Charlat

**Affiliations:** Université de Lyon, Université Lyon 1, CNRS, UMR 5558, Laboratoire de Biométrie et Biologie Evolutive43 boulevard du 11 novembre 1918, Villeurbanne, F-69622, France

**Keywords:** Bioinfomatics/phyloinfomatics, molecular evolution, phylogenetic theory and methods, phylogeography

## Abstract

Inferring phylogenetic relationships between closely related taxa can be hindered by three factors: (1) the lack of informative molecular variation at short evolutionary timescale; (2) the lack of established markers in poorly studied taxa; and (3) the potential phylogenetic conflicts among different genomic regions due to incomplete lineage sorting or introgression. In this context, Restriction site Associated DNA sequencing (RAD-seq) seems promising as this technique can generate sequence data from numerous DNA fragments scattered throughout the genome, from a large number of samples, and without preliminary knowledge on the taxa under study. However, divergence beyond the within-species level will necessarily reduce the number of conserved and non-duplicated restriction sites, and therefore the number of loci usable for phylogenetic inference. Here, we assess the suitability of RAD-seq for phylogeny using a simulated experiment on the 12 *Drosophila* genomes, with divergence times ranging from 5 to 63 million years. These simulations show that RAD-seq allows the recovery of the known *Drosophila* phylogeny with strong statistical support, even for relatively ancient nodes. Notably, this conclusion is robust to the potentially confounding effects of sequencing errors, heterozygosity, and low coverage. We further show that clustering RAD-seq data using the BLASTN and SiLiX programs significantly improves the recovery of orthologous RAD loci compared with previously proposed approaches, especially for distantly related species. This study therefore validates the view that RAD sequencing is a powerful tool for phylogenetic inference.

## Introduction

Resolution of phylogenies between closely related species can be problematic for a number of reasons. First, due to incomplete lineage sorting and introgression, different loci might trace different evolutionary histories. In addition, most nuclear markers lack resolution at short evolutionary scales. And finally, in poorly studied taxa, molecular markers might not have been developed. In this context, Restriction site Associated DNA sequencing (RAD-seq) appears as a promising approach. This technique relies on the high throughput sequencing of genomic regions flanking restriction sites (Baird et al. 2008; Davey et al. 2010; McCormack et al. 2013; Rowe et al. 2011). It thus generates numerous homologous markers, scattered throughout genomes, potentially from hundreds of specimens in a single sequencing run. Moreover, if the precise number of loci is not critical, this approach is potentially universal, as it does not require preliminary knowledge on the taxa under study (Baxter et al. 2011).

The RAD-seq technique was initially designed to generate informative molecular variation within species, and has repeatedly proved its efficiency for this purpose (e.g., Emerson et al. 2010; Hohenlohe et al. 2010). However, the utilization of RAD-seq to compare genomes from different species can potentially be hindered by a number of caveats. Specifically, the following conditions must be fulfilled for RAD-seq to be suitable for phylogenetic inference: (1) enough restriction sites must be conserved between species; (2) the flanking regions must be sufficiently conserved for homology to be detectable by sequence similarity; and (3) the resulting alignments must contain enough phylogenetic signal.

Recent studies, published as this article was in preparation, concur to suggest that these conditions are often fulfilled (Rubin et al. 2012; Wagner et al. 2013). Here, we use a simulated RAD-seq experiment on the 12 *Drosophila* genomes (Clark et al. 2007) to assess if this is the case at various degrees of molecular divergence, and to optimize the procedure of RAD data analysis for phylogeny. Consistently with Rubin et al. (2012), we were able to recover and align enough sequences from orthologous loci to reconstruct the known (whole-genome-based) phylogeny (Clark et al. 2007), with good statistical support. We further show here that sequence clustering based on the BLASTN and SiLiX programs significantly improves the recovery of orthologous RAD loci. This study therefore validates and reinforces RAD-seq as a powerful tool for phylogenetic inference.

## Divergence time and RAD loci conservation

The number of RAD loci potentially usable to compare two specimens is the number of restriction sites conserved in their genomes, which is expected to decrease as divergence time increases. We used the 12 *Drosophila* genomes to establish the relationship between genome divergence and restriction sites conservation. Complete genomes and pairwise alignments of the *D. melanogaster* genome with each of the 11 others were downloaded from http://genome.ucsc.edu/. Conserved target sites of the restriction enzyme Sbf1 were counted in each pairwise alignment. This enzyme is one of the most commonly used in RAD-seq studies because its 8 bp, GC-rich recognition site is rare enough in genomes to maximize sequencing coverage for each locus.

**Figure 1:**
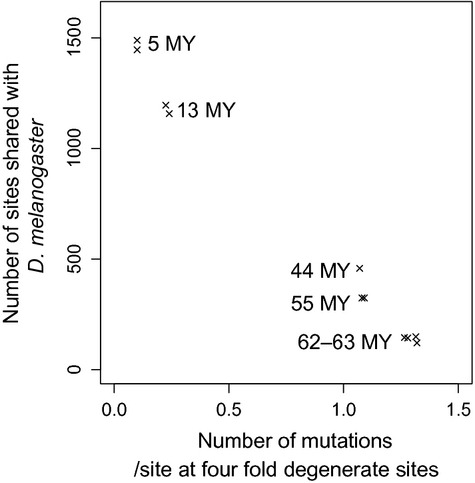
Relationship between molecular divergence at fourfold degenerate sites (*Drosophila* consortium, 2007) and Sbf1 restriction site conservation. Note: genomic regions aligned more than once against the *D. melanogaster* genome were excluded. Numbers next to crosses indicate divergence time.

Figure 1 shows the relationship between restriction site conservation and sequence divergence (measured at fourfold degenerated sites of codons), with divergence times ranging from 5.4 to 63 My (Tamura et al. 2004). The genome of *D. melanogaster* contains 2,948 sites, 49.1% and 50.5% of which are conserved in its two closest relatives *D. simulans* and *D. sechellia*. After 63 My of divergence, *D. melanogaster,* respectively, shares 4.9, 4.8, and 5.1% of its restriction sites with *D. grimshawi*, *D. mojavensis,* and *D. virilis*, which represent 145, 142, and 149 restriction sites. Notably, these estimates are conservative as the 2,948 restriction sites in *D. melanogaster* are counted from its complete genome, while conserved sites are only detected in the aligned genomic regions.

## How many RAD tags are usable for phylogeny?

The above results suggest that more than 100 Sbf1 cut sites are conserved between species having diverged for 63 My (Fig. 1). However, for the genomic regions flanking these sites to be usable for phylogenetic inference, sequences must be sufficiently conserved for homology to be detected. Moreover, paralogous loci may confound phylogenetic analysis and should therefore be excluded.

Illumina sequencing reactions yield 101 bp or 51 bp long reads. Here, we focus on the longest reads, which maximize the potential phylogenetic signal (see below for a discussion on this issue). Each read starts with a barcode sequence identifying a sample (up to 8 bp long) and the 8 bp restriction site followed by 85 bp of usable data. We thus extracted 85 bp in 5′ and 3′ of each restriction site to set up the list of RAD sequences existing in each *Drosophila* genome. To identify sets of homologous sequences among the 12 species, we used a two-step procedure: first, we performed all-against-all BLASTN comparisons (Altschul et al. 1990); and second, we analyzed these results with the SiLiX software (Miele et al. 2011), to cluster sequences sharing a minimum level of sequence identity over a minimal length (see below for a discussion on the optimal parameter values). As can be seen in Table 1, the proportion of orthologous RAD tag pairs retrieved by BLASTN and SiLiX (i.e., gathered in the same cluster) decreases with divergence time from 99% (between *D. melanogaster* and *D. simulans*, 5.4 My of divergence) to 49% (*D. melanogaster* and *D. wilistoni*, 63 My of divergence).

**Table 1:** Number of known and retrieved orthologous RAD tags in each species pair. “Orthologous tags”: total number of orthologous RAD tags present in pairwise alignments (*D. melanogaster* vs. each of the 11 other species). “Retrieved orthologous tags”: proportion of orthologous tags clustered by SiLiX. “In clusters including paralogs”: proportion of the retrieved orthologous tags clustered in groups containing more than one locus. Loci are defined here based on genome sequences (see main text). Node depth from Tamura et al. (2004)

Species pair *D. melanogaster*	Node depth (My)	Orthologous tags	Retrieved orthologous tags (%)	In clusters including paralogs (%)
*D.sechellia*	5.4	2978	99	5
*D.simulans*	5.4	2892	99	4
*D.erecta*	12.6	2390	97	3
*D.yakuba*	12.8	2314	97	8
*D.ananassae*	44.2	916	68	9
*D.persimilis*	54.9	648	65	9
*D.pseudoobscura*	54.9	648	66	9
*D.wilistoni*	62.2	242	49	6
*D.grimshawi*	62.9	290	60	8
*D.virilis*	62.9	286	59	5
*D.mojavensis*	62.9	298	59	8

Restriction sites located in repeated regions may be problematic for phylogenetic analysis and should therefore be identified and discarded. For every pair of species, we thus identified clusters containing more than one locus from at least one of the two species. The exclusion of these clusters leads to a loss of less than 10% of the RAD tags (Table 1). Among the clusters containing only single copy loci, we observed 2% of “false positives,” that is, clusters containing loci that are not considered as orthologous based on whole-genome alignments.

## Phylogenetic inference from simulated RAD-seq data

The above estimates rely on genome alignments for the identification of clusters containing paralogous RAD loci. In a true RAD-seq experiment, the “locus” definition would be solely based on an initial step of clustering of reads from each specimen. At this step, recently duplicated RAD tags may be mistakenly grouped into a single “locus” and too divergent alleles from the same locus can be mistakenly identified as two different loci. To estimate more realistically which proportion of the data would be usable for phylogenetic inference, we therefore simulated sequencing and “intra-specimen” clustering steps in our analysis. This simulation also allows us to test the impact of sequencing errors, heterozygosity, and coverage variation.

To assess the effect of heterozygosity on data analysis, a second haploid genome was simulated for each species using random mutations of the sequenced genome, to produce a 5% average distance between homologous alleles (the upper bound of realistic polymorphism values). RAD sequencing was then simulated by randomly sampling reads from the list of all possible RAD loci from diploid genomes, with a 10× mean coverage per locus, which was shown to cover 99% of all RAD loci at least once (see below). For each sampled read, sequencing errors were added with a uniform 1% error rate, that is, the upper bound of Illumina error rate estimates (Illumina technical support, Glenn 2011). Intra-individual clustering of the reads was performed using the ustacks program (Catchen et al. 2011). This program forms “stacks” (groups of strictly identical reads within individuals) and clusters similar stacks to form putative loci, with a user-defined maximum number of differences between stacks within a locus. Ustacks finally aggregates secondary reads (that were not initially placed in a stack) into existing stacks, to better estimate coverage. To maximize the number of retrieved heterozygous loci, we allowed up to 13 differences between stacks within a locus. We observed that this high value did not increase the number of inferred loci containing paralogous reads (3% of the loci). We also allowed up to nine mismatches to cluster secondary reads to putative loci. Using such parameters, 11.6% of the loci were mistakenly split by ustacks. This shows that a high level of heterozygosity in the data does not hamper the clustering of reads within loci (only few reads from the same locus are mistakenly clustered into different loci).

Orthologs were then searched with BLASTN and SiLiX as described above, using as input the consensus of each ustacks locus. We observe that 1.1% of the clusters that are known to contain paralogs (based on genome alignments) are not identified as such in the simulated experiment, as they were mistakenly merged by ustacks in a single locus. These correspond to very recent duplication events that should not confound the phylogenetic signal, unless many of these duplications are anterior to the latest speciation event.

**Figure 2:**
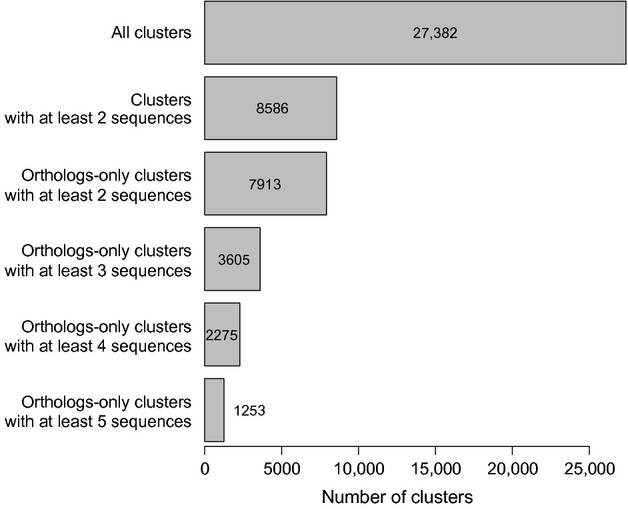
Results of SiLiX clustering of RAD sequences from the 12 *Drosophila* genomes.

**Figure 3:**
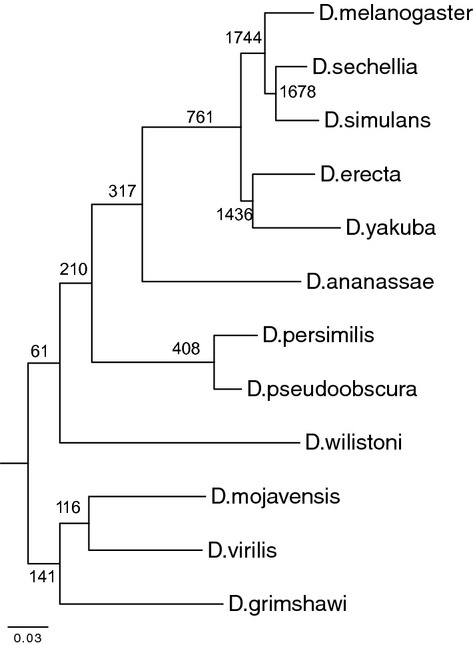
RAD-seq-based phylogeny of the 12 *Drosophila* species, based on 100-bp-long RAD-seq reads, inferred by maximum likelihood using PhyML 3.0. under a GTR+ G substitution model, using the concatenated alignments from orthologous-only clusters containing at least four sequences. Bootstrap values (100 replicates) were equal to 100% on every node. We indicate the number of informative loci at each node (shared by at least one species on each side of the bifurcation and at least one outgroup).

For each cluster, sequences were aligned with Muscle v3.8.31 (Edgar 2004) using default parameters. We selected and concatenated the 2,275 topologically informative alignments (i.e., containing sequences from at least four different species, Fig. 2), adding gap sequences to represent missing orthologs. The proportion of gaps in the alignment varied from 19.2% for *D. simulans* to 94.9% for *D. wilistoni* (Table 2), which is expected considering the topology of the phylogenetic tree (the probability to recover at least three orthologs for a given locus is higher for species that have many close relatives in the set of sampled species). A maximum likelihood phylogeny was built using PhyML (Guindon et al. 2010). Bootstrap support was calculated with 100 replicates. The expected phylogenetic relationships among the 12 *Drosophila* species, established from whole-genome comparisons (Clark et al. 2007), were correctly recovered with strong bootstrap supports (Fig. 3). Because the number of RAD loci conserved between genomes decreases with divergence time, the number of loci that can be used to infer phylogeny tends to decrease for the deepest branches of the tree (Fig. 3). However, there remains enough phylogenetic signal to infer the correct topology with high confidence, even for the most ancient branches.

**Table 2:** Percentage of gaps for each species in the concatenated alignment, after exclusion of loci present in less than four species

Species	Percentage of gaps in the concatenated alignment (%)
*D.melanogaster*	24.6
*D.sechellia*	20.4
*D.simulans*	19.2
*D.erecta*	25.5
*D.yakuba*	23.4
*D.ananassae*	75.5
*D.persimilis*	80.4
*D.pseudoobscura*	80.0
*D.wilistoni*	94.9
*D.grimshawi*	91.8
*D.virilis*	91.5
*D.mojavensis*	91.1

## Practical issues: sequencing coverage, number of specimens, and read length

In a typical RAD-seq experiment, DNA samples from several individuals are tagged with molecular identifiers and multiplexed in the same flow cell lane. The average sequencing coverage per locus per individual is given by the following formula:


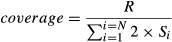


where *R* is the total number of reads, *N* the number of individuals included in the library preparation, and *S*_*i*_ the number of restriction sites in the genome of individual *i*. Increasing the pool size allows a decrease in sequencing cost per sample, but leads to a lower coverage per locus, which should affect the number of loci that can be recovered. The proportion of loci that was sequenced at least once in the in silico experiment follows a Poisson distribution: with 5× or higher coverage, more than 99% of the expected loci are sequenced at least once; this proportion drops to 95% with a 3× coverage. However, RAD loci represented by only one read are not identified as valid RAD loci by ustacks (Emerson et al. 2010; Morris et al. 2011). Using two as the minimum coverage to create a stack in ustacks parameters, the proportion of loci for which at least one allele was recovered after the intra-individual clustering step is 88.3% for a 10× mean coverage, which was sufficient in our simulations to recover the expected phylogeny. The fact that only 88% of the loci are recovered (although 99.95% are sequenced at least twice) is due to the high levels of polymorphism and sequencing error used in our simulations. Although those values are conservative, we would recommend an increased coverage.

Notably, a significant proportion of restriction sites fall in recently duplicated regions, which reduces the number of RAD loci actually usable for phylogeny. For example, the reference sequence (haploid) of the *D. melanogaster* genome contains 2,948 restriction sites, that is, 5,896 potential RAD tags. Intra-individual clustering of reads from this genome using ustacks yields 4,296 clusters, 3% of which contain reads from more than one locus. This small proportion of clusters containing recently duplicated regions represents a substantial proportion of all reads (25.3%). Thus, 74.7% of reads actually fall in non-recently duplicated regions.

The 12 *Drosophila* species analyzed here contain on average 2,308 Sbf1 restriction sites per genome. Sbf1 is a rare-cutter enzyme because of its 8-bp-long and GC-rich restriction site. It is expected to yield among the lowest number of restriction sites per genome. This property is valuable for studies where a large number of specimens matters more than a large number of loci per genome. For example, with an Illumina Hiseq 2000 flow cell lane (*R* = 1.5 × 10^8^ reads), and for a targeted coverage of 10×, it would, in theory, be possible to pool DNA samples from up to 1000 specimens (for a genome size comparable to that of Drosophila). Of course, a number of issues would potentially lower the coverage for some loci or some specimens (e.g., genome size, GC content, DNA template concentration, and quality). Targeting a 50× mean coverage would allow the analysis of 200 specimens in one flow cell lane, and would be robust to a 10-fold variation in coverage between loci or specimens.

With existing sequencing technologies, it is also possible to increase coverage or reduce sequencing cost by decreasing the length of the reads. For example, Illumina sequencing runs can yield 101-bp- or 51-bp-long reads. We investigated whether 51 bp RAD-seq reads could be used for phylogenetic studies. We extracted 35 bp sequences on both side of each restriction sites and performed the in silico RAD-seq experiment and phylogenetic analysis as described above (see supplementary material for details). Overall, the topology shows only one error (the position of *D. wilistoni* is incorrect). Recent nodes (younger than 45 My) were resolved with high support within the *Melanogaster* group (*D. melanogaster, D. simulans, D. sechellia, D. yakuba, D. erecta,* and *D. ananassae*), but bootstrap values were low (<80%) for some deep nodes. This poor support for deep nodes suggests that short reads should only be used to resolve short-scale phylogenies.

## Orthology inference: SiLiX versus uclust

As we were finishing this manuscript, Rubin et al. (2012) published a comparable analysis and also reached the conclusion that RAD-seq can be a useful tool for phylogenetic inference. One notable difference between the two studies is the use of different procedures to identify orthologous loci: uclust (Edgar 2010) versus SiLiX.

**Figure 4:**
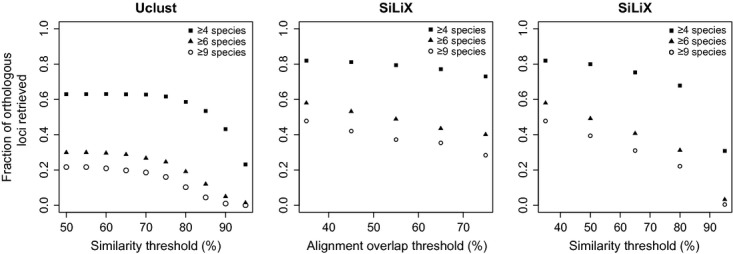
Proportion of known orthologous sequences retrieved by SiLiX or uclust, for different clustering parameters. Results are shown for clusters containing at least 4, 6, or 9 species. Note the x-axes are not the same in all figures. For SiLiX, the values of two parameters are tested: the minimum overlap threshold (on the second figure, where the minimum identity parameter is set at 0.35), and the identity threshold (on the third figure, where the minimum overlap is set at 0.35).

In an attempt to optimize this crucial step, we conducted a comparison of the two methods using the same data set and various parameter values. We estimated the efficiency of orthology detection by calculating the proportion of orthologous RAD loci (known from the whole-genome alignment) that were correctly recovered (i.e., known orthologs gathered in the same cluster, without inclusion of any paralog). It should be noticed that this definition of “efficiency” reflects the combined effect of sensitivity (the ability to gather orthologous sequences) and specificity (the ability to exclude paralogs). To assess the effect of evolutionary distance on clustering efficiency, we computed this measure for clusters shared by at least 4, 6, or 9 species (Fig. 4).

The first step of the SiLiX procedure consists in comparing all sequences against each other with BLASTN. To avoid the detection of spurious sequence similarities, we allowed the filtering of low complexity sequences (parameter F = T, which is set by default in BLASTN) and we set the *E*-value parameter to 10^−4^ (i.e., stringent enough to avoid the detection of similarities between non-homologous sequences). In the second step, SiLiX takes into account two parameters to cluster two sequences in a family: the fraction of their length covered by the BLASTN alignment (alignment overlap) and their sequence similarity (in the aligned region). The clustering of uclust is based on a global alignment, and requires one single parameter (the minimum percentage of identity over the entire sequence length). As expected, for both methods, the efficiency of orthology detection decreases with increasing evolutionary distance (compare in Fig. 4 the efficiency for RAD loci shared by 4, 6, or 9 taxa). As noted by Rubin et al. (2012), the efficiency of uclust decreases when the sequence similarity threshold is set to very high value (≥80%), and we observed the same trend for SiLiX (Fig. 4). For SiLiX, the efficiency also tends to decrease with increasing alignment overlap threshold. We therefore recommend using relatively permissive thresholds (sequence identity ≥35%, and sequence overlap ≥35%). Interestingly, we observed that SiLiX was significantly more efficient than uclust, especially for clusters including some relatively distant homologs: for loci shared by at least nine species, SiLiX was two times more efficient than uclust (44.7% vs. 21.6% efficiency). In principle, the main interest of uclust is that it is extremely fast, because it does not require an exhaustive comparison of all sequences against each other's. However, given the number of sequences that have to be compared in a typical RAD-seq experiment (several thousands of loci for several hundreds of specimens), calculation time is not limiting. For example, in our analyses, the clustering of 55,324 sequences took 7 s with uclust versus 3 min for BLAST + SiLiX (on a single Macbook Pro with a 2.9 GHz processor and 8 Gb of memory). Moreover, one disadvantage of uclust is that its result depends on the sequence that is used as a seed to initiate the clustering. Hence, uclust clustering has to be replicated many times with different seeds to check results consistency. As the BLAST + SiLiX method is exhaustive, appears to be more efficient, and does not require to be replicated with different seeds, we argue that it should be preferred to uclust.

## Conclusion

Our phylogenetic analysis using simulated RAD-seq data suggests that this method is suitable for interspecific comparisons, even for relatively large genetic divergences (1.3 substitutions per site, which corresponds to 63 My for the *Drosophila* clade). Enough non-duplicated restriction sites were conserved between species and sequence conservation between orthologous RAD tags was sufficient to detect homology for a large number of loci. Finally, the recovered alignments contained enough phylogenetic information to yield strongly supported phylogenies. It should be noted that in our simulations, we did not incorporate incomplete lineage sorting. However, empirical data suggest that RAD-seq produces data from enough loci to overcome this problem (Wagner et al. 2013).

This study further indicates that the SiLiX clustering program is more efficient than uclust to identify orthologous RAD sequences, especially for distantly related species, for which this feature is most critical. In addition, we observed that RAD-seq-based phylogenies are robust to sequencing errors and high polymorphism values. In practice, we recommend to target a 50× coverage, which is sufficient to sample 99% of all RAD alleles at least once, even with a 10-fold coverage variation between specimens, and to avoid the use of short reads (50 bp), which would lead to significant loss of phylogenetic signal for deep nodes. Overall, this study validates and reinforces RAD-seq as a powerful tool for phylogenetic inference.

## Methods

We provide below program versions and parameter values used in this study, when not specified in the main text:

Ustacks (Catchen et al. 2011)
ustacks -t fasta –f file.fasta -r -m 2 –N 9 –M 13.
BLASTN: blastall 2.2.25 (Altschul et al. 1990)
blastall -i file.fasta -d file.fasta -p blastn -o file.blastn -W 11 -b 10000 -v 10000 -z 1000000 -e 1e-4 -m 8
SiLiX (Miele et al. 2011)
silix file.fasta file.blastn -r 0.35 –i 0.35
Muscle v3.8.31 (Edgar 2004)
Default parameters
PhyML 3.0. (Guindon et al. 2010)
phyml -i file -d nt -b 100 -m GTR.

## Supporting Information

**Figure S1.** Phylogeny of the 12 *Drosophila* species based on 50-bp-long RAD-seq reads, inferred by maximum likelihood using PhyML 3.0. under a GTR + G substitution model, using the concatenated alignments from orthologous-only clusters containing at least four sequences. Bootstrap values (100 replicates) are indicated in italics. Rad-seq data were simulated with 1% sequencing errors, but without polymorphism. Notice that bootstrap supports for several deep nodes are low and that the placement of *D. wilistoni* is incorrect. Non-italic numbers indicate the number of informative loci for each node (shared by at least one species on each side of the bifurcation and at least one outgroup).
