# Supplementary material for: Is RAD-seq suitable for phylogenetic inference? An in silico assessment and optimization
Source: Ecol Evol. 2013 Feb 27;3(4):846–52. doi: 10.1002/ece3.512 (PMC3631399; doi:10.1002/ece3.512)
Supplement: Supplementary file 1 [file ece30003-0846-SD1.docx]

**Figure S1. Phylogeny of the 12 *Drosophila* species based on** **50 bp long RAD-seq reads,** inferred by maximum likelihood using *PhyML 3.0.* under a GTR + G substitution model, using the concatenated alignments from orthologous-only clusters containing at least four sequences. Bootstrap values (100 replicates) are indicated in italics. Rad-seq data was simulated with 1% sequencing errors but without polymorphism. Notice that bootstrap supports for several deep nodes are low and that the placement of *D. wilistoni* is incorrect. Non-italic numbers indicate the number of informative loci for each node (shared by at least one species on each side of the bifurcation and at least one outgroup).
